# Role of gender in the treatment experiences of people with an eating disorder: a metasynthesis

**DOI:** 10.1186/s40337-018-0207-1

**Published:** 2018-08-13

**Authors:** Priyanka Thapliyal, Phillipa Hay, Janet Conti

**Affiliations:** 1 Translational Health Research Institute (THRI), School of Medicine, Western Sydney University, Sydney, NSW Australia; 2 School of Social Sciences and Psychology, Western Sydney University, Sydney, NSW Australia

**Keywords:** Eating disorders, Gender, Treatment, Experiences

## Abstract

**Background:**

Traditionally perceived as a disorder of women, Eating Disorders (EDs) are known to have impacts on people irrespective of their gender. This study is designed to synthesise the available qualitative research studies to more broadly understand the diverse experiences of ED and their treatment, specifically in relationship to issues of gender.

**Methods:**

The methodology involved a systematic search and quality appraisal of the literature published after 1980 using terms that aimed to represent the primary concepts of “role of gender” and “treatment experiences” and “eating disorders”. Nine qualitative studies met the inclusion criteria. Meta-themes were inductively generated through a synthesis of data across themes from the relevant included papers.

**Results:**

Analysis of data was constructed around three meta-themes, each with subthemes. The first meta-theme “Out of sight, out of mind” depicted the experience of gender issues that were marginalised in treatment. More specifically for transgender people, when gender issues were ignored by treatment providers, this frequently led to non-disclosure of their gender identity. Furthermore, men were less likely to be assessed for an eating disorder and within this context; diagnosis of an ED and referral to specialist treatment was frequently hindered. The second meta-theme “Lack of literacy among health care providers” focused on issues related to misdiagnosis of EDs, and the question of whether this was related to a lack of health literacy amongst health professionals. The final theme “Pathways into treatment that address stigma and other barriers” highlighted the need for the development of future treatment interventions address the complex social reality of the experiencing person, including questions of gender.

**Conclusion:**

Gender issues impact upon the ED experience and require broader consideration in the development and evaluation of ED treatment interventions, including the further development of gender-informed interventions.

**Trial registration:**

Protocol registered on PROSPERO 2017 CRD42017082616.

**Electronic supplementary material:**

The online version of this article (10.1186/s40337-018-0207-1) contains supplementary material, which is available to authorized users.

## Plain English summary

This meta-synthesis aims to more richly understand issues related to gender in EDs and their treatment across relevant qualitative research studies. For the purpose, 9 qualitative research studies that represented concepts of “role of gender”, “treatment experiences” and “eating disorders” were analysed and meta-themes across the studies were constructed.

Results are presented in the form of three meta-themes that consist of a number of subthemes. The first meta-theme highlighted the significance of gender issues in the ED experience and some of the impacts of this on the ED treatment experience, particularly for transgender people and men, The second meta-theme constructed the gap in addressing gender issues in treatments as a lack of health literacy amongst health providers in the assessment and treatment for an ED. Consistent with the perspectives of the experiencing person, the final meta-theme emphasised the need for a gender-informed and gender-specific treatment for eating disorders; this focus may ultimately result in better treatment outcomes.

## Background

Eating disorders (EDs) are long known to be much more frequent in women than men and thus have been typically constructed as a woman’s disorder. A meta-analysis [1] of community surveys reported the lifetime, 12-month, and 4-week prevalence of EDs among females as 4.2-, 2.6-, and 3.2- fold the corresponding prevalence in males. ED presentations differ across gender as men are more likely to report overeating whereas women are more likely to endorse loss of control while eating [2]. Women have been found to have higher scores for ED behaviours such as drive for thinness, bulimia and body dissatisfaction compared to men [3]. Results from a comparative study [4] have also suggested that men were more likely to present with less severe ED pathology (weight, shape and eating concerns and dietary restraint) than women. However, it is unclear whether this was due to a true difference in symptomatology or to biases inherent in the measures towards thinness rather than muscular/larger bodies, which are more favoured by men [5]. Cross-sectional studies also have found that men with EDs exhibit high rates of psychiatric co-morbidity [6–8], a later age of onset [9] and a history of premorbid overweight [10] compared to women with EDs.

### Gender and the under identification of experiences as ED

Men are less likely to seek ED treatment services and are also less likely to be diagnosed with an ED if they do seek help for psychological difficulties. In Australia, only 13.6% of men with EDs have ever sought ED treatment [11]. Men have reported greater difficulty in disclosing ED problems as the admission of being a male with an ED does not fit with the societal perceptions of EDs as occurring only in women [12]. Other barriers identified for seeking professional treatment for an ED for both men and women have included stigma and feelings of shame and fear [13].One study found the main sex difference in the experience of stigma being the extent to which sufferers are made to feel “less of a women” versus ‘less of a man’ [14]. In addition to the findings that men are reluctant to seek help [15] there exists a lack of awareness among clinicians and under diagnosis of EDs in men [12, 16]. This is despite similar symptomatology [17, 18] similar treatment responses [19–21] and similar outcomes [17] among men and women with EDs.

There nevertheless exists a paucity of research comparing treatment outcomes between men and women. One of the few published studies that has compared men and women’s responses to a day hospital treatment program reported no significant differences [22]. On the other hand, a study that compared the response rate to cognitive behavioural therapy between a group of 131 men and 131 women between 1998 and 2015 found that male ED patients who completed treatment (specifically BN and OSFED) were more likely to achieve full remission than women with the same diagnosis [23]. In contrast, men who engage in treatment with significantly low body weight, co-morbidity and little family support, like women, have been found to experience greater difficulty in achieving a positive treatment outcome [24].

A greater understanding of factors that contribute to difficulties in engaging in care for EDs is needed. Some have argued that interventions need to focus on male specific issues such as weight history, gender orientation, compulsive exercise, body image and dynamics of depression and shame [25]. Whilst there seems to is an argument for the provision of tailored treatment interventions for men [21], or “male-friendly” services [26], there is no substantive research or recommendations for other groups, such as transgender people. The perspectives and experiences of the person have also tended to be obscured by treatment outcome studies that focus on researcher selected variables including weight restoration and ED symptomatology. Additionally, there are notably few studies that have focussed on the treatment experiences of men [21] and even fewer that focus on the experiences of ED and their treatment for transgender individuals.

Furthermore, there is lack of consensus about relevance of gender per se in treatment [27]. This is despite the push for more of a focus on feminist issues in ED treatments by women [28] and the development of feminist therapies [29].

### Current review

Despite a number of literature reviews [12, 26, 30, 31] on gender and EDs, there currently exists no meta-syntheses of qualitative research studies in this area. Additionally, there has been limited consideration within these reviews of the potentially diverse treatment experiences of people who identify as Lesbian, Gay, Bisexual, Transgender and Intersex (LGBTI). This paper is designed to address this gap through synthesising the available qualitative research on gender issues in EDs and their treatment and, through a richer understanding of the diversity of ED, inform future treatment interventions.

## Methods

The present study employed meta-ethnography methodology [32] and followed ENTREQ statement (Enhancing Transparency in Reporting the synthesis of Qualitative research) [33]. ENTREQ consists of 21 items grouped into five main domains: introduction, methods and methodology, literature search and selection, appraisal, and synthesis of findings.

### Databases and search methodology

A pre-planned systematic search of the literature was conducted in October and November, 2017 using the Electronic Database (Scopus), Psych Info, Pub Med (Medline), grey literature database (digital thesis) and generic web searches (Google Scholar). Subsequently, a hand-search of the reference lists from the selected papers was followed. The search terms are presented in Additional file 1: Search terms “Eating disorders” OR “Anorexia” OR “Bulimia” OR “Binge eating” AND “Treatment” OR “Therapy” AND “Gender” OR “Men” OR “Male” OR “Women” OR “Female” AND “Qualitative”. These terms aimed to represent the primary concepts of “role of gender” and “treatment experiences” and “eating disorders” with PUBYEAR> 1980.

#### Study selection

The initial database search returned 45 abstracts after removing duplicates. This included studies that were located through hand-searching the reference lists in the potential papers and provided further key papers and so on iteratively to achieve a comprehensive range of articles. The primary researcher (PT) then screened the abstracts and excluded studies that did not address the research aim. The second stage of the study selection process involved examining each of the 45 articles and considering those that satisfied the following inclusion criteria:

1. Journal peer reviewed article, thesis and Pro Quest;
2. Articles published from1980–2017;
3. Participants had an ED according to current diagnostic schemes (Diagnostic & Statistical Manual of Mental Disorders (DSM) or International Statistical Classification of Disease and Related Health Problems (ICD));
4. The study addressed treatment experience and included any gender (male, female or transgender);
5. Used a qualitative study methodology;
6. The study addressed some aspects of the role of gender or sexual orientation in the treatment for people with an ED.

The increase in number of ED cases and publications occurred in early to mid-eighties and this coincided with establishment of the *International Journal of Eating Disorders* in 1982 and development of contemporary treatments. Therefore, articles published from 1980 were included.

**Fig. 1:**
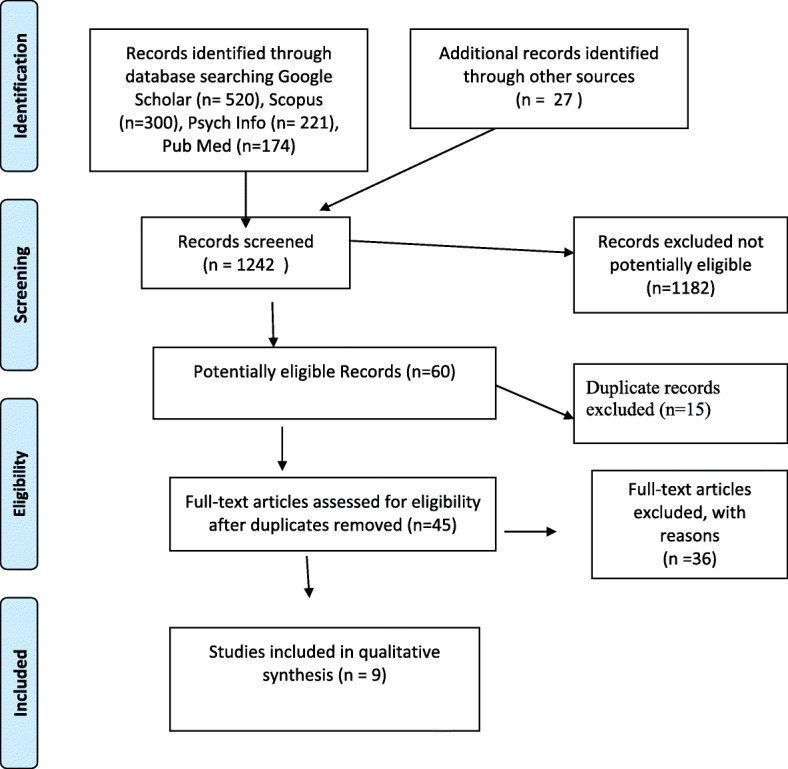
Prisma Flow Diagram

Forty-five studies met these criteria 1–4 and included for further review by two researchers (PT and PH). Studies were excluded that were not qualitative and/or excluded gender issues in treatment (details are provided in the Additional file 2: Table S2). A summary of the excluded studies grouped by the primary reason of exclusion is provided in additional file 2: List of studies excluded from the review with exclusion reason. This process resulted in a total of 9 studies that met inclusion criteria into the metasynthesis. Figure 1 is the PRISMA flow diagram.

#### Coding of studies

Each of the 9 included studies were coded using a rating sheet according to the following characteristics: Author name, year published, country (location of study), age of participants (age range or mean), and sample size, gender (male, female, and transgender).

#### Analysis strategy

The studies employed a qualitative methodology to examine gender issues as relevant to the ED experience and its treatment. Studies were excluded if they were not published in English, not about EDs and were not a study of treatment experience. All the included studies were assessed for quality by all authors based on the Relevance, Appropriateness, Transparency and Soundness (RATS) [34] and Critical Appraisal Skills Programme (CASP) [35] criteria namely: 1) relevance, where the research question was explicit and linked to the existing knowledge base, 2) methods were described and justified, 3) there was transparency in regards to selection, recruitment, data collection, role of researchers/investigators, and ethics, and 4) there was rigor of analysis and reporting.

Meta-themes were inductively generated through a synthesis of data across themes from the included papers. We followed the method described in Shaw [36] where all three authors:

1. Read and coded the papers separately with the intention of generating diverse codes through these multiple perspectives with the aim of generating “thick description” ([37], pg.6) of the data and enhance the validity of data interpretation and analysis.
2. These diverse codes were collaboratively constructed, through group discussion and consensus on interpretative understanding [38] into a table of themes as reported in the primary paper (first-order constructs);
3. Together authors translated first-order constructs, that located the subjective experiences of participants across studies, into second order constructs or meta-themes that interpreted collective experiences within the context of a larger structure [39] and provided a comparison of concepts between studies; and
4. Presented findings as a series of meta-themes supported by quotes from the original papers.

## Results

### Study characteristics

The characteristics of the qualitative studies about role of gender in the treatment experiences of ED are detailed in Table 1.

**Table 1:** Characteristics of included studies

S.N	Author, Year, Country	Participants	Eating Disorder diagnoses	Study question	Study design and transparency	Rigor of analysis and reporting
1	Dearden &Mulgrew (2013), Australia	1) representatives of organizations, 2) practitioners, and 3) 5 men clients aged 22–58 years, long-standing ED issues of 4–19 years	Two with formal diagnosis of AN and BED	Research question clear based on systematic review	Purposive sampling, followed by snowballing, males were self-selected by invitation form health care organization, general-inductive approach, open-ended written self-report single author thematic analysis	Quality mixed, unclear role of researchers and ethics, analytic approach described, quotes used and triangulation (3 sources), saturation not reported, limitations discussed (small sample and use written survey)
2	Raisanen& Hunt (2014), UK	Men (*n* = 10) age 17-25 years 7-Heterosexual, 3- gay	AN, BN& EDNOS	Research question clear	Qualitative interviews.	Qualitative interpretative approach, use of quotes. Themes and subtheme discussed in detail.
3	Duffy et al. (2016), USA	Transgender (*n* = 84)	AN, BN, BED & EDNOS	Clear research question.	Online questionnaire	Inductive thematic analysis
4	Robinson et al. (2013), UK	Men (*n* = 8)	AN, BN & EDNOS	Research question clear.	Semi structured interviews.	Interpretative phenomenological analysis
5	Beer and Wren (2012) UK- England Specialist services	Men (*n* = 9), aged 21–42 years	All but most AN	Research question clear and based on systematic review	Interviews, interpretive phenomenological analysis, secondary report of unpublished thesis thus transparency unclear	Quality unclear, design and analytic approach not reported in publication (book chapter)
6	Drummond & Murray (2002), Australia	Men (*n* = 8) age unclear	AN& BN	Research question clear and based on systematic review	In-depth qualitative interview, provided for flexibility.	Inductive approach, repeated examination of the data to identify common themes in relation to the phenomenon being researched.
7	Su Holmes (2016) UK	Women (*n* = 15), age 19–45 years, 13-heterosexual, 1-bisexual, 1- bi/pansexual	AN, BN, BED	Clear research question	One-to-one interview	Thematic discourse analysis,
8	Holmes et al. (2017), UK	Women (*n* = 7), age 19–51 years	AN	Clear research question	Semi-structured individual interview	Thematic Discourse analysis
9	Crenshaw (1998) Thesis	Women (*N* = 10) aged 27–59 years of 40 participants in a feminist therapy	AN = 2, BN purging = 2, BN non purging = 6	Clear research question	Purposive sampling; Semi-structured individual interview	Constant comparative method of data analysis

*AN* anorexia nervosa, *BN* bulimia nervosa,*BED* Binge eating disorder

**Table 2:** Translation of themes related to gender issues in treatment for an ED across the primary papers

THEMES	SUB-THEMES	PAPER-ORIGIN
1. Out of sight out of mind	a) So bloomin’ obvious: Gender issues “erased” or undervalued in treatment	Duffy et al., 2017 [40],Holmes, 2016 [28], Holmes, 2017 [44], Robinson et al., 2013 [12].
	b) Decision to not disclose gender identity (Transgender group)	Duffy et al., 2016 [40].
2. Health literacy regarding gender issues amongst health care providers	a) Under or misdiagnosed in men.	Dearden &Mulgrew, 2013 [26], Drummond, 2002 [41], Raisanen& Hunt, 2014 [43].
	b) Health literacy specific to gender	Duffy et al., 2017 [40], Holmes, 2016 [28], Robinson et al., 2013 [12]
	c) Does gender really matter in treatment?	Crenshaw [29], Drummond, 2002 [41], Holmes, 2017 [44], Holmes, 2016 [28], Robinson et al., 2013 [12]
3. Creating Pathways into treatment that address stigma and other barriers	a) A women’s problem? Addressing stigma for men	De Beer & Wren [42], Holmes 2016 [28], Raisanen & Hunt, 2014 [43]
	b) Gender informed treatment	Crenshaw [29], Dearden & Mulgrew, 2013 [26], Duffy et al., 2016 [40], Holmes 2016 [28], Raisanen & Hunt, 2014 [43].

#### Methodologies employed

All the studies were conducted using known qualitative methods with the qualitative data being generated by interview (seven studies), self-written report (one study) and online questionnaire (one study).

#### Year and location of studies

The studies that met selection criteria were published between the years 1999 and 2017 with the majority conducted in the United Kingdom (*n* = 5) followed by Australia (*n* = 2) and United States (*n* = 2).

### Sample and participant characteristics

#### Sample size

Apart from the online survey study [40] that had a larger sample size (*n* = 84), all the other qualitative studies had sample sizes between 5 to 15 participants.

#### Participant age

In all but one study [41], the age of participants were documented, with participants reported to be between 16 to 58 years.

#### Gender

In the studies included in the metasynthesis, 5 had only male participants [12, 26, 41–43], 3 had only female participants and focused on gender issues in treatment [28, 29, 44] and one study [40] explored the treatment experiences of transgender that had female, male and non-binary (not categorically either gender) participants.

#### Type of eating disorder

The studies comprised of participants diagnosed with AN (*n* = 2), AN, BN, BED and EDNOS (*n* = 2), AN, BN and BED (*n* = 1), only AN and BN (*n* = 4), and two studies did not specify participants’ ED diagnosis.

#### Sexuality

The studies included people who identified as heterosexual, bisexual, Bi/pansexual female (*n* = 2), only female where sexual orientation was unknown (*n* = 1), only heterosexual male (*n* = 2), hetero and gay males (*n* = 3), and female, male and non-binary participants (*n* = 1).

### Synthesis of themes

**Fig. 2:**
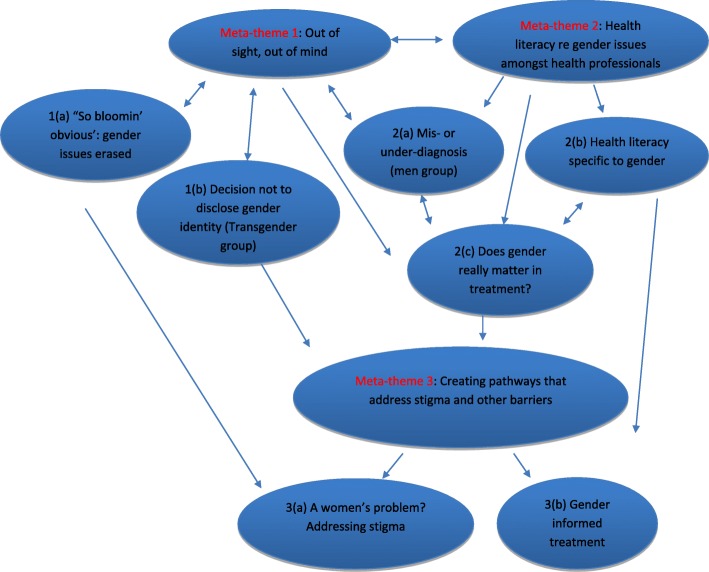
Meta-themes of the experience of gender in eating disorder treatment

The identified themes related to gender issues in treatments were translated and presented in Table 2. This metasynthesis generated a series of interconnected meta-themes that highlighted the interrelationship between gender and ED treatment experiences (Fig. 2). The implications for clinical practice are then discussed.

#### Theme 1: Out of sight out of mind

Questions of gender identity were consistently, although likely to be unintentionally, marginalised by treatment service providers for women [28], men [12] and transgender people [40]. These issues were argued, by those who identified them, as relevant and significant to the ED experience and their frequent omission in treatment was highlighted across the studies.

**a) “So bloomin’ obvious”: Gender issues “erased” or undervalued in treatment**

Of the two studies that explored gender issues for women [28, 44], the majority of women experienced gender as not specifically addressed in their treatment.

*EXTRACT 1*

*“It would have been a whole lot easier if these [feminist] perspectives] were used in my treatment…. It always felt like me against the system which is really isolating…I would have seen that it wasn’t just me being crazy…. The idea of eating disorders being a problem of society I actually found really reassuring…. rather than just you’ve gotta sort yourself out and fit back into the society structure that we’ve got.”* (P8) [28].

For participant 8 [28] the lack of focus on gender issues in treatment was not neutral in its effects and contributed to a sense of isolation. Additionally, the absence of focus on the societal context within which EDs develop recruited her into the idea of herself as “just me being crazy” and an individualistic perspective of having to “sort yourself out”.

Gender issues were positioned by the women as integral to their experience and they struggled to understand this omission in ED treatments, with one participant arguing that their inclusion should be *“so bloomin’ obvious”* (P8) [28]. When addressed in therapy, however, there was a tendency in one study [45] to accommodate gender onto pre-existing treatment interventions and rendered these issues as only superficially addressed: “*I was just told, um, to try and not notice images of bodies*. *.*. *It’s a bit ridiculous really*.” (P9) [28].

In the only study with transgender people [40], around 60% (*n* = 50) of the participants disclosed their gender identity to their clinicians.

*EXTRACT 2a*

“*Every primary care provider that I have interacted with simply sidesteps or ignores my articulation of a trans identity. It’s as if the gesture never happened*” (P) [40].

*EXTRACT 2b*

*“*Since *coming out, my identity has been erased in treatment because clinicians and therapists aren’t comfortable with a transgender individual who does not identify as male or female*” (P) [40].

Participants who disclosed their gender identity to therapists were most frequently concerned by the experience of their gender identity being ignored by practitioners (for example, Extract 2a). The power of this therapist response was highlighted by another participant for whom an important dimension of their selfhood was erased (Extract 2b). An overarching theme in this study highlighted a parallel process between the struggle to “come out” as transgender and the struggle for health professionals to engage with this dimension of their selfhood. This ran counter to their yearning for acceptance of their gender identity from the health professionals who treated them.

One study into men’s experiences of treatment found that being a solitary male in a group therapy intervention was experienced and interpreted as purposeful exclusion, even if this may not have been the intention of the facilitator or group members.

*EXTRACT 3*

*“[As the only male] you become aware of people holding back or you being purposely excluded, or people saying ‘maybe it’s best if you’re not in this group”* (Bill) [12].

Within this stretch of text, the impacts of being the solitary male was a sense of himself as an outsider to the group with his exclusion being understood as what’s in the “best” interest of the group. This indicates the power of being marginalised through gender difference and that there are real effects of gender being ignored in ED treatment interventions.

**b) Decision to not disclose gender identity (transgender group)**

In the one study into transgender people’s experience of ED treatment, 40% (*n* = 28) of the participants reported that they decided not to disclose their gender identity to treatment providers [40].

*EXTRACT 4a*

*“I have stopped telling therapists, doctors, and groups that I don’t identify as a woman. It makes them uncomfortable and alienates me. Lying is easier*” (P) [40].

*EXTRACT 4b*

*“I did not discuss my issues around gender identity when I was in the treatment program, and I was treated as though I were a male patient”* (P) [40].

Centred on this “choice” not to disclose their gender identity was an effort to avoid stigma and discrimination that they had previously experienced through specific past negative experiences with health care providers, including that their disclosure “makes them [health professionals] feel uncomfortable” (P) [40], or simple logistics (e.g., treatment centre that treated only women). Furthermore, this concealment of their gender identity contributed to further marginalisation (“alienates me”) [40], the sense of themselves as “lying” (Extract 4a) and their identity being assumed to be that of the gender with which they did not identify (Extract 4b).

#### Theme 2: Health literacy regarding gender issues amongst health care providers

Implicit in the frequent absence of gender issues being addressed in therapeutic contexts was the question of the health literacy. This meta-theme encompasses the varied contexts within which when the participants across these studies experienced gender issues as marginalised in their treatment.

**a) Mis- or under diagnosis in men**

Men’s experiences of help-seeking indicated that to qualify for treatment and/or their struggles to be taken seriously, they needed to disclose and present with more serious patterns of ED symptomatology. Two of the 5 studies that researched men’s treatment experiences, found that 30% [43] to40% [26] of men were misdiagnosed with either all the men [26],or a proportion of them [43], being given other mental health diagnoses that included mood disorders and anxiety.

*EXTRACT 5a*

*“The one GP I went to, back when it was first starting and I was a somewhat healthy weight still, dismissed my concerns as” “just stress*” (P) [26].

*EXTRACT 5b*

*“You haven’t got bulimia, you're just depressed, I’m probably quite confident in saying that was probably because you know I was male, you know I didn’t live up to the stereotype of being young and female*” (P3) [43].

These men argued that their distress was minimised and “dismissed” by the health professionals who treated them. Two of the 5 men in Dearden & Mulgrew’s study [26] highlighted how their distress was measured by the extent of their weight loss (Extract 5a) and one man’s experience of minimisation was signified through his use of quotation marks as “just stress”.

Another participant’s (Extract 5b) [43] experience of minimisation of their symptoms by a health professional was understood as him not fitting the gendered “stereotype” of the sort of person diagnosed with an ED. Instead his experience was positioned and minimised as “just depressed”, which ruled out a more complex formulation that took into consideration the interrelationship between ED symptoms, under nutrition and low mood [46].

The issue of being under diagnosed contributed to further distress in 4 of 16 men across 2 studies [41, 43] through a heightening of ED symptomatology and associated health risks.

*EXTRACT 6a*

*“I didn't like drinking too much overnight. [However,] I did start to restrict how much water I was having and stuff like that. Actually that idea came into my head from one of the psychiatrists down at the clinic. He asked me if I was restricting my fluid intake. And up until then, I wasn't restricting my fluid intake. I didn't really figure water was going to do too much. He basically told me straight out, “We can't do too much for you here at our clinic. I don't know why you were sent here...because you're not at the critical level.” So I figured if I'm going to get to this critical level I may as well start restricting my water intake*” (P) [41].

*EXTRACT 6b*

*“I didn’t say anything. I was dumbfounded, like I couldn’t believe a physician would say something like that, because I just thought, you know, if there’s nothing physically wrong but something’s happening surely that means there’s something psychological. And if it’s to the extent where I’ve lost three stone at this point, you know, shouldn’t I be getting referred. … basically I walked outside and punched a wall and broke my knuckle because I was so angry. That he’d sat there and called me weak and blah blahblah [Precisely] to” “man up” and “Not be weak but be strong and deal with the problem*” (P5) [43].

The perceived response of being denied treatment because of not being at a “critical level” (Extract 6a) was for one man an incitement to compete for the diagnosis and treatment through an escalation of ED behaviours that included restriction of fluids. The interaction between gender and the social practice of competitiveness may have profound implications for how men engage or do not engage in ED treatments. This may become increasingly dangerous in contexts where health care resources are scarce and health professionals inadvertently do not consider the possibility or extent of men’s ED symptoms and distress. In extract 6a, the participant’s negotiation of social power and status was to engage more intensively in ED practices to prove that he had an ED and qualified for treatment. On the other hand, participant 5’s [43] response of anger signified an identity violation [47] that was built on a masculine discourse where ED behaviours in a man were assumed to be a sign of weakness rather than distress (“he called me weak”; Extract 6b). The gendering of the ED experience builds further the assumption that the experience of an eating disorder, like gendered constructions of women [48], is a sign of weakness in men.

**b) Health literacy specific to gender**

The lack of health literacy amongst health care practitioners was reported across the diverse ED experiences of transgender [40] people, men [12] and women [28]. This sub-theme encapsulated the emotional distress experienced by the participants in these studies when gendered issues were ignored.

*EXTRACT 7a*

*“I have been regularly misgendered over the course of my treatment. It's kind of par for the course by now, but it's still really upsetting. Even my therapist doesn't really understand how to use my pronouns and has referred me to as “girl” and “daughter”, despite the fact that I've come out to her*” (P) [40].

*EXTRACT 7b*

*“I can’t help but …wonder how my life might have been different …if anyone who saw me in the course of my treatment had been able to recognize my gender dysphoria and inform me that there were ways of addressing my extreme discomfort with my post-pubertal body other than starving myself ”* (P) [40].

*EXTRACT 7d*

*“My therapist is excellent in her work, but it seems she does not understand non-binary genders*” (P) [40].

In the only study that explored the experiences of transgender people [40], 11% (*n* = 9) described the experience of being misgendered by the clinicians who treated them, that was after disclosing their gender identity and 17% (*n* = 14) of participants argued that clinicians need to be more informed about gender-specific issues. This experience of being misgendered was not neutral in its effects and contributed to further distress (for example, extract 7a). The marginalisation of their transgender experiences and how these were inextricably intertwined with a range of other experiences, including the ED, was experienced as central in their struggles to progress in treatment (Extracts 7b). Even when a therapist was experienced as “excellent” (Extract 7c), there was evidence of an unexamined world view that was underpinned by the dominance of both heterosexual [49] and dualistic gender norms. The absence of scope to focus on their gender identity meant that an important aspect of their embodied subjectivity and lived experience (including their experience of an ED) was (inadvertently) obscured by therapeutic processes.

The difficulty in finding ED treatment services that had scope to tailor services to their needs and preferences, appeared to be a crucial problem for transgender participants in this study [40]. In addition, the lack of comprehensive ED treatment services ameliorated the efforts to seek holistic treatment for a trans, rape survivor (Extract 7c). Within this extract, this participant challenged and criticized the present health care system as having failed them.

Likewise, studies into women’s ED experiences highlighted the implications of a lack of focus on gender issues on these women’s sense of identity.

*EXTRACT 8*

*“When I was reading [Orbach’s Hunger Strike] . . . I tried bringing it up with the psychiatrist . . . She would just look at me like I was barmy – like that was more madness coming from me” . . . I was quite confused about it but it was like “OK let’s put [the book] . . . away again: that’s just [name removed] being crazy again . . .*”(P5) [28]

The understanding of her ED experience within its societal context enabled participant 5 [28] to resist the pathologisation of herself as “crazy” and assisted her navigating her identity within a society that prizes the thin female body [50]. On the other hand, the dominance of an individualistic disorder discourse over a feminist perspective on EDs meant that when she raised feminist issues in treatment, she interpreted her therapist’s response as confirming her identity “crazy” person.

Although the experience of body image issues and their relationship to the EDs maybe divergent across genders, a consistent theme across these experiences was a lack of recognition of gender issues in therapies that “pigeonhole” people into the dominant construction of an ED as a woman’s disorder. This was identified as particularly problematic in the non-dominant groups of men and transgender people.

*EXTRACT 9a*

*"The groups I’d be sitting in on ... on the ward with all the women ... body image things, they really didn’t bear any relation to ... the issues that I had"* (Bill) [12].

*EXTRACT 9b*

*"In spite of my identification, "professionals" tend to fall back on essentialist notions: "you are a man." I am simply not interested in educating professionals about my gender or identity; it's the one space where I do not have the energy left to do so"* (P) [40]

Despite these diverse experiences of one man (Extract 9a) and a transgender woman (Extract 9b), both these people faced a sense of alienation in their respective treatment interventions. Being the only male in a treatment program, Bill experienced himself as an outsider whose issues as a man were left unaddressed. The transgender participant (Extract 9b) who no longer identified as a man felt frustrated and abandoned by health professionals due to repetitive assumption of his identity as “a man”.

**c)Does gender really matter in treatment?**

All the eight studies highlighted the diverse views of participants on whether gender issues mattered in treatment and there was a lack of consensus about its relevance for inclusion in the understanding of EDs and their treatment. For example, in one of the 5 studies on men [12], there were contradictory views both within and between the men’s narratives about whether to raise the questions of gender in treatment or not.

*EXTRACT 10a*

*“I think it’s pretty much gender-excluding as a disorder. The reasons why you get there are probably slightly different but in the end all roads lead to Rome* [...] *the gender part is not significant but no ... too many people with like mind and that mind are deranged”* (Bill) [12].

*EXTRACT 10b*

*“Everyone is different, and ultimately every case is different, and although people suffering from bulimia have got the same thing, it’s probably for a number of different reasons*” (Greg) [12].

*EXTRACT 10c*

*“Perpetuation of maintenance of their disorders as a feminized illness” (P)* [41].

Bill [12] struggled between his experience as an outsider in a program focused on women’s issues (extract 9a) and his position that “gender part is not significant” (extract 10a). In taking up an individualistic discourse to understand the ED experience (“the mind is deranged” (Bill, extract 10a) and “everyone is different” (Greg, extract 10b)), the men’s accounts were troubled on the question of whether gender was relevant in the ED experience and its treatment. On the other hand, one out of 6 men in Drummond & Murray study’s [41] critiqued ED treatments as being built on the construction of EDs as a “feminized illness” (extract 10c).

Underpinning these diverse perspectives is the tension between addressing the unique issues faced by an individual, yet also understanding an individual and their ED experience within their social context. Likewise, the women in these studies also expressed diverse opinions on whether gender was relevant or not to their experience of an ED and its treatment.

*EXTRACT 11a*

*“People that aren’t female get eating disorders as well… I think that men are also held to quite high standards when it comes to exercise and things like that so…I don’t know; I don’t think eating disorders really are a feminist issue”* (P4) [44].

*EXTRACT 11b*

*“Feminism…. I like the idea of women having rights… but then I think it can also go borderline, you know, women are great, men aren’t… When we ask for more and more and more through feminism, I think it’s borderline sexist” *(P2) [44].

These women were also troubled by the question of whether EDs are appropriately understood and treated as a feminist issue (Extract 11a) or whether by arguing for their inclusion in treatment, they were being “sexist” themselves (Extracts11b). Although gendered constructions structure reality in ways that are frequently outside a person’s conscious awareness, one woman positioned therapy that addressed these complex issues as tapping into “deeper… more subtle” dimensions of her lived experience, including “*expectations of men and women around food… and weight loss*” (Extract 11c) (P7) [44]. Although women in one study [33] experienced a notable absence of gender issues being addressed in their ED treatment, another study found women’s experiences of a feminist group ED intervention to be beneficial [29] (see below Theme 3b for further detail). This intervention focused on consciousness raising [29] thereby drawing on feminist perspectives as transformative to the intervention, rather than merely accommodative of gender [45] as an “add on” to treatment as usual.

#### Theme 3: Creating pathways into treatment that address stigma and other barriers

**a) A women’s problem? Addressing stigma**

The construction of eating disorders as a “women’s disorder” presented a challenge to men’s masculine identity, particularly as the feminising of a disorder is frequently associated with weakness (see subtheme 2a). Therefore, the application of this diagnostic category to men was troubled, particularly in one study [42] where the men reported feeling emasculated through stigma associated with an eating disorder diagnosis.

*EXTRACT 12a*

*“It can feel like an admission of being less than male*” (P) [42].

*EXTRACT 12b*

*“I didn’t like being pushed around*.” (P) [42].

The men’s responses to an ED diagnosis and its treatment were diverse. Assumed in a masculine discourse area sense of authority, competitiveness and physical strength [51]. Therefore within this context it was hardly surprising that these men struggled to initiate help-seeking for an ED. Help-seeking may be understood as a fear of losing autonomy through giving up control and a mistrust of giving over control over to others [44]. An ED diagnosis was also associated with self-stigma of having a “female illness” as one of the men associated an ED with being “*less than male”* (Extract 12a) and, in the context of an inpatient admission, another man associated care with control and being “pushed around” in treatment (extract 12b). The experience of being “pushed around” in treatment sat in contrast to one woman’s experiences of being “pushed” to talk about her experiences in the context of a feminist-informed intervention.

*EXTRACT 13*

*“Right we need to talk about this, and [she] really pushed me…It…was then that somebody actually clicked that there was more going on than people had picked up on”* (P3) [28].

This woman recounted how being “pushed” by her psychologist after a near successful suicide attempt was a helpful therapeutic intervention for her at that time. This juxtaposition between this man and woman’s experiences highlights how gender is intertwined with power and the ways this is negotiated within the therapeutic relationship and the treatment intervention.

*EXTRACT 14*

*“I did make contact with my doctor…I just said that what I had done and what had happened…he talked through the whole process [er] with me and explained that, the very serious side effects to doing that to myself. [Um]Obviously encouraging me you know for it not to continue doing what I can. [Er] He was happy to refer me [um] to someone, but you know that was my choice*” (P7) [43].

Although the recurrent issues for men across the studies were either misdiagnosis or a lack of gender specific treatment, in the above extract one man talked about what was helpful in his treatment experience was clear communication and a sense of personal agency in choosing his treatment pathways.

**b) Gender informed treatment**

A recurrent theme across women’s inpatient treatment experiences was the extent by which they experienced themselves as having voice in their treatment.

*EXTRACT 15*

*“It is only once you learnt that kind of submissive femininity where you don’t protest and you don’t seek “excessive” independence or your own views that you’d be kind of granted the right to speak again, although on, like, really limited terms*” (P12) [28].

This woman highlighted how although she was “granted the right to speak” (extract 15), this was on the terms of a submissive voice that was unquestioning of their treatment [28]. Implicit in this woman’s experience is the power of the health professionals to confine the terms of speaking to the dominant treatment discourse. Experienced as authoritarian and harsh, one participant recounted how she was withheld one-to-one therapy until she reached a baseline weight [28].

In contrast, the study of a therapeutic intervention that aimed to raise a feminist consciousness was experienced as supportive with the 7 participants describing an initial anxiety which progressed to ‘acceptance’ and an enhanced sense of self-worth, empowerment and increased confidence.

*EXTRACT 16a*

*“It’s impacted my self-confidence as a woman. In fact, a lot of my life, I would say earlier in my life I would say to people, “don’t respond to me as a woman, respond to me as a person.” I’m responding to you not as a man but as a person. To try to neutralize the playing field. But the point is, I am a woman. And so coming to grips with that—and now I make no excuses for it. I claim it. And it takes two people to fight. It takes two people to really engage in an energetic disagreement, so I just don’t engage. I state my point of view and . . .off we go. It’s been—it really has helped me claim my space with pride. Not with fear, but pride”.* (Mary) [29].

*EXTRACT 16b*

*“Well, I guess that the biggest thing that I learned in this program about feminist consciousness that I’m using is knowing that I can explore all possibilities of sexuality. That’s the biggest thing that I have learned”* (Katie) [29].

The women in this study described greater acceptance and ownership of both their gender and sexual orientation (extract 16a) as well as a sense of personal agency to choose their sexual orientation, which for Katie was experienced as a sense of expansiveness of her experiences of her sexuality (extract 16b). Leila spoke of choosing to have lesbian friends for the first time *“who’ve told me that I am attractive, sensual, and I don’t feel put off or frightened by that.”* [29] and Mary spoke of choosing to hold a friend’s hand who happened to be a woman and not being afraid to do so.

The need for a gender-inclusive treatment was also argued by studies focused on men’s ED treatment experiences, with a number of men arguing that the available information was either gender-blind or gender inappropriate.

*EXTRACT 17a*

*“Like you hear the side effects of having an eating disorder on like women. Like they can become infertile and stuff like that, but I’ve never seen any for men.” So I like went and was like, “look well what are they for men? ‘Cos like that I could like have a side effect and wouldn’t know*” (P1) [43].

*EXTRACT 17b*

*“The only information I got was [er] a scare sheet basically. It was this was going to happen if you keep going. Basically the big one that they circled was, ‘Oh you won’t be able to be sexually active’ for men. And that was the biggest thing” *(P5) [43].

*EXTRACT 17c*

*“If we had more male friendly health care system more men might talk about eating issues” *(P) [26].

For the 9 men in Raisanen & Hunt ‘s study [43], the lack of male-specific information had a range of impacts including the obscuring of information related to the physical effects of an ED for men (Extract 17a)or the antithesis of this, where this information was perceived by one man as a ‘a scare sheet’ that highlighted the possibility of impotence or lack of sex drive (extract 17b). The concerns regarding gender specific treatment for men ran across these studies with one respondent in the Dearden & Mulgrew study [26] who believed that having a more *“male friendly”* health care system might provide a context for men to voice their experiences (extract 17c) .

*EXTRACT 18*

*“It would be reassuring to see programs which include some sort of reference to their inclusivity of transgender/gender-diverse people”* (P) [44].

The lack of appropriate ED treatments led to a reluctance for some participants who identified as transgender to reach out for fear of receiving inadequate or inappropriate care. For others who sought help, the process of trying to identify an appropriate treatment centre was experienced as stressful. Nevertheless, there was still sense of optimism among the transgender participants to have inclusive treatment centres in future.

## Discussion

The present study synthesised qualitative research studies in order to understand the experience of gender in the experience of an ED and its treatment across diverse groups of people. Meta-themes captured the consistent and diverse experiences across these studies where gender issues were experienced as marginalised by therapists, treatment teams and treatment interventions for women, transgender and men. When gender issues were addressed in ED treatments, participant experiences indicated that the dominant therapeutic response was to accommodate these issues onto pre-existing individualistic formulations and treatment models that construct the ED as a disorder that exists within [52]. That was, rather than gender issues being addressed in ways that transformed these approaches [53].

For the women, this was experienced as their voice being filtered and constrained within the treatment context where they were permitted to speak, but mainly within the terms of the dominant individualistic disorder discourse [45] thereby leaving intact this discourse intact [53]. Within these contexts, interventions were reported to only superficially address the real issues that these women faced that were intertwined with their gender. One study, however, found that when gender was directly addressed in a group context that the women experienced greater ownership and acceptance of their gender and sexuality and understood their experience in the context of a broader socio cultural perspective.

Men repeatedly spoke of under diagnosis and/or requiring severe ED symptom patterns to qualify for specialised ED treatments. Although this issue of symptom severity as a gateway to ED treatment services is not unique to men [51], there continues to exist a question as to whether men’s ED symptoms and distress need to be more severe and of greater risk before they are noticed by health professionals as eligible for an ED diagnosis and its treatment [54]. This issue of requiring more intense levels of ED symptomatology for men with an EDs to be taken seriously for treatment, echoed the prevalent notion that EDs are a “female illness” [12, 17, 55]. Furthermore, the use of physical diagnostics, including BMI and body weight as central criteria for ED diagnosis and assessment of ED severity were perceived by a number of men as questionable. In addition to under diagnosis, when men’s distress was located within the ED discourse, they frequently reported a lack of access to tailored treatment interventions that addressed the unique issues faced by men.

For transgender people, the lack of health literacy and tailored interventions that acknowledged their gender identity and how this interrelated with their ED experience was a substantive finding, albeit from the only research study in this population that met criteria for inclusion in this metasynthesis. Transgender participants faced presumptions and bewilderment from clinicians about how to respond to their gender identity and provide a holistic treatment intervention that could handle the complexity of the interrelationship between the ED experience and their identity as transgender. These experiences of transgender people that questioned the mental health professionals ‘competence in treating individuals identifying as transgender was of major concern and resonated with previous studies [56, 57]. Participant’s sought acknowledgement and acceptance of their gender identity by the therapist and reportedly felt ignored and marginalised. The predominance of negative responses is striking and participants’ experiences indicated that this added to their distress. The non-disclosure of gender-identity could have influenced the treatment efficacy as concealment of one’s identity impedes necessary aspects (for e.g., trust, mutual respect, and therapeutic alliance) of treatment [58].

Implicit in the marginalisation of gender issues in ED treatments was the meta-theme of an inadequacy of health literacy in relation to gender issues for women, men and transgender people. Across these studies there was consistent evidence that ignoring gender in therapeutic interventions was not neutral in its effects. However, the question of whether or not therapists should raise and address gender in treatments was also troubled as a number of participants across these studies also argued that treatments should not focus on EDs as a feminist issue [59]. There was only one woman who expressed a preference for a specific feminist approach and one (supportive) study of a therapy that was transformed by feminist issues in ED. This is likely to reflect deficiencies in the availability of and the evidence for, feminist therapies for ED [60]. Furthermore, it was noteworthy that for some women, being recruited into a submissive position during ED treatment acted to reinforce their perceptions that this is normal and appropriate for their gender.

The unique needs and preferences for men in ED treatments were highlighted where men talked of their isolation in programs that were tailored to predominantly treat women, lacked ED health information specific to EDs in men and frequently failed to focus on the unique issues of what it means for a man to be diagnosed with a problem that is predominantly associated with women. For example, in light of the conflicted assumptions embedded in a sense of masculine identity that contributes to gender role conflict and emasculation in men [55, 61] the ED experience may contribute to a flawed sense of identity that a man who experiences an ED is “less than a male” [42]. Studies reported [41, 62, 63] that men expressed a wish for their *maleness* to be recognised in treatment. Engagement is ED treatment was experienced as difficult for these men because of the unique challenges faced by them, particularly around societal gender norms and masculinity that are associated with control and physical strength [64]. Thus there is the risk of a dissonance in the therapeutic relationship where men are ambivalent about losing autonomy and the therapist’s view of them as a patient in need of treatment. It is therefore, important to train practitioners in the identification of EDs in men, including in interventions that give men therapeutic opportunities to examine and question gender norms rather than inadvertently applying them to themselves (for example, through the practice of narrative therapy [65]). It is pertinent to know where and how men position themselves on masculine discourses related to strength, power and control because this has implications for whether and how they engage in ED treatment services. Providing room for men to have a voice in therapy and context to challenge some of these masculine norms around strength control and power that act as restraints to help-seeking may assist them to engage earlier and more meaningfully in treatment interventions for ED.

A concerning finding is that few of the participants in the studies reported having had a positive experience in relation to the ways gender was addressed in ED treatments. This gap in treatment services is likely to be a significant barrier to care and has significant implications for clinical practice and future research.

### Implications for clinical practice and future research

The present metasynthesis raises a number of issues that are of clinical importance in the implementation of gender issues in ED treatments. A notable finding relates to the importance of increasing health literacy amongst health professionals, which resonates with other research that has identified the need to address the skills of health professionals in ED assessment and treatment interventions [66, 67]. There also exists a need to address some of the assumptions, biases and negative stereotypes, particularly in relation to people who identify as transgender. The significant absence of health professional literacy related to the unique issues faced by those who identify as transgender risked in heightening these peoples’ distress. There exists a clear need for health professionals to address their often unintentional biases and negative assumptions about people who identify as transgender; for example, through reflective practice in supervision [68].

The interaction between gender and the social practice of competitiveness may have profound implications for how men engage or do not engage in ED treatments. This may become increasingly dangerous in contexts where health care resources are scarce and health professionals inadvertently do not understand the extent of men’s symptoms and distress. Courtenay [69] has argued that “social practices that undermine men’s health are often signifiers of masculinity and instruments that men use in the negotiation of social power and status” (p.1385). A proportion of the men across these studies negotiated social power and status through more intensively engaging in ED practices to prove to those who treated them, that they had an ED and to qualify for treatment. Previous studies in gender and ED have argued [55, 70, 71] that women have been the focus of information around EDs epidemiology, symptoms, assessment and treatment, leaving a paucity of relevant knowledge for other populations (males and transgender) to inform and tailor treatment interventions. One example is the use of physical diagnostics (e.g. body weight and Body Mass Index-BMI) for assessment and weight as the important criteria for ED diagnosis needs to be amended because of the risk this poses to the under-identification and diagnosis of EDs in men who typically lie on the elevated side because of body musculature.

Though the relationship between gender and ED is not a linear, causal relationship, the experience places an individual in certain sociocultural niche, where he/she may be more prone to specific risk factors such as media influences [72]. Therefore, recognition and tailored treatments of EDs that are transformed by gendered perspectives, including those of the non-dominant and vulnerable groups (men and transgender) needs more detailed consideration.

#### Limitations and strengths

A strength of this metasynthesis is the rigorous qualitative search for papers and with coding by 3 authors before deriving themes from the relevant studies. As metasynthesis do not have access to the original data, sources of bias in the original primary studies (for example misinterpretation of participants’ voice) can introduce bias in the synthesis [73]. The reasonably smaller number of studies included in the review could be considered a limitation; however, this has allowed exploration and analysis of interview data in these papers in greater detail to enrich understanding. That there was only one study on ED experiences of transgender people highlights the extent of marginalisation of this group. Additionally, all the studies included were in English, leading to a possibility of missing other relevant articles published in non-English language.

## Conclusion

This metasynthesis highlights the inadequacies in ED treatments in addressing issues related to gender amongst diverse gender populations. How identity is negotiated in the context of the lived experience of an ED is embodied and this metasynthesis provides evidence that gender plays a significant role in this embodiment. Therefore, the conscious or inadvertent ignoring or marginalising of questions and issues related to gender in ED treatments has and will continue to have inevitable implications for the processes and extent by which people engage in ED treatments. Greater consideration and inclusion of gendered perspectives in ED identification and the transformation of ED treatments holds scope for more significant and meaningful positive outcomes for those with lived experience.

## Additional file

Additional file 1: **Table S1.** Search Term Strategy. (DOCX 31 kb)

Additional file 2: **Table S2.** Details of excluded papers for metasynthesis. (DOCX 50 kb)

## Abbreviations

AN: Anorexia Nervosa
BED: Binge Eating Disorder
BMI: Body Mass Index
BN: Bulimia Nervosa
CASP: Critical Appraisal Skills Programme
DSM: Diagnostic and Statistical Manual of Mental Disorders
EDNOS: Eating Disorders Not Otherwise Specified
EDs: Eating Disorders
ENTREQ: Enhancing Transparency in Reporting the synthesis of Qualitative research
ICD: International Statistical Classification of Diseases and Related Health Problems
LGBTI: Lesbian, Gay, Bisexual, Transgender and Intersex
OS/UFED: Other Specified or Unspecified Eating Disorders
RATS: Relevance, Appropriateness, Transparency and Soundness
