# Supplementary material for: Role of gender in the treatment experiences of people with an eating disorder: a metasynthesis
Source: J Eat Disord. 2018 Aug 13;6:18. doi: 10.1186/s40337-018-0207-1 (PMC6088416; doi:10.1186/s40337-018-0207-1)
Supplement: Supplementary file 1 — Table S1. Search Term Strategy. (DOCX 31 kb) [file 40337_2018_207_MOESM1_ESM.docx]

Additional file 1

Additional file 1: TableS1 Search Term Strategy

| **DATABASE** | **SEARCH TERMS** | **URL** |
| --- | --- | --- |
| Pub Med | (“Gender" OR "sex" OR "male” OR “men” OR “women” OR “female”) AND (“treatment OR therapy”) AND (“eating disorder" OR “anorexia” OR “bulimia” OR “binge eating”) AND (“qualitative”). | https://www.ncbi.nlm.nih.gov/pubmed/?term=(%E2%80%9CGender%22+OR+%22sex%22+OR+%22male%E2%80%9D+OR+%E2%80%9Cmen%E2%80%9D+OR+%E2%80%9Cwomen%E2%80%9D+OR+%E2%80%9Cfemale%E2%80%9D)+AND+(%E2%80%9Ctreatment+OR+therapy%E2%80%9D)+AND+(%E2%80%9Ceating+disorder%22+OR+%E2%80%9Canorexia%E2%80%9D+OR+%E2%80%9Cbulimia%E2%80%9D+OR+%E2%80%9Cbinge+eating%E2%80%9D)+AND+(%E2%80%9Cqualitative%E2%80%9D). |
| Psych Info | (“Gender" OR "sex" OR "male” OR “men” OR “women” OR “female”) AND (“treatment OR therapy”) AND (“eating disorder" OR “anorexia” OR “bulimia” OR “binge eating”) AND (“qualitative”). | http://web.b.ebscohost.com.ezproxy.uws.edu.au/ehost/results?vid=3&sid=9be5b45e-93ba-422f-945c-9576e231bb48%40sessionmgr103&bquery=XX+%22binge%22%5b100%5d+OR+XX+%22bulimia%22%5b99%5d+OR+XX+%22anorexia%22%5b94%5d+OR+XX+%22eating%22%5b81%5d+OR+XX+%22men%22%5b57%5d+OR+XX+%22gender%22%5b52%5d+OR+XX+%22women%22%5b49%5d+OR+XX+%22qualitative%22%5b48%5d+OR+XX+%22sex%22%5b45%5d+OR+XX+%22disorder%22%5b40%5d+OR+XX+%22therapy%22%5b39%5d+OR+XX+%22treatment%22%5b30%5d+OR+XX+%22female%22%5b22%5d+OR+XX+%22male%22%5b21%5d&bdata=JmRiPXBzeWgmdHlwZT0xJnNpdGU9ZWhvc3QtbGl2ZSZzY29wZT1zaXRl |
| Google Scholar | (“Gender" OR "sex" OR "male” OR “men” OR “women” OR “female”) AND (“treatment OR therapy”) AND (“eating disorder" OR “anorexia” OR “bulimia” OR “binge eating”) AND (“qualitative”). | https://scholar.google.com.au/scholar?as_sdt=0,5&q=(%E2%80%9CGender%22+OR+%22sex%22+OR+%22male%E2%80%9D+OR+%E2%80%9Cmen%E2%80%9D+OR+%E2%80%9Cwomen%E2%80%9D+OR+%E2%80%9Cfemale%E2%80%9D)+AND+(%E2%80%9Ctreatment+OR+therapy%E2%80%9D)+AND+(%E2%80%9Ceating+disorder%22+OR+%E2%80%9Canorexia%E2%80%9D+OR+%E2%80%9Cbulimia%E2%80%9D+OR+%E2%80%9Cbinge+eating%E2%80%9D)+AND+(%E2%80%9Cqualitative%E2%80%9D).+&hl=en&as_ylo=1980&as_yhi=2017 |
| Scopus | (“Gender" OR "sex" OR "male” OR “men” OR “women” OR “female”) AND (“treatment OR therapy”) AND (“eating disorder" OR “anorexia” OR “bulimia” OR “binge eating”) AND (“qualitative”). | <https://www.scopus.com/results/results.uri?sort=plf-f&src=s&st1=gender+OR+sex++OR+male+OR+men+OR++female+OR++women&st2=treatment+or+therapy&nlo=&nlr=&nls=&sid=b3a9993610c2950cab651089bf2acdd6&sot=b&sdt=b&sl=212&s=%28TITLE-ABS-KEY%28gender+OR+sex++OR+male+OR+men+OR++female+OR++women%29+AND+TITLE-ABS-KEY%28treatment+or+therapy%29+AND+TITLE-ABS-KEY%28eating+disorder+or+anorexia+or+bulimia+or+binge+eating%29+AND+TITLE-ABS-KEY%28qualitative%29%29&cl=t&offset=201&origin=resultslist&ss=plf-f&ws=r-f&ps=r-f&cs=r-f&cc=10&txGid=08f135c50f987268ffd367461978715b> |
