# Supplementary material for: Role of gender in the treatment experiences of people with an eating disorder: a metasynthesis
Source: J Eat Disord. 2018 Aug 13;6:18. doi: 10.1186/s40337-018-0207-1 (PMC6088416; doi:10.1186/s40337-018-0207-1)
Supplement: Supplementary file 2 — Table S2. Details of excluded papers for metasynthesis. (DOCX 50 kb) [file 40337_2018_207_MOESM2_ESM.docx]

Additional file 2

Additional file 2: Table S2

| **SN** | **TITLE** | **AUTHOR** | **YEAR** | **REASON FOR EXCLUSION** |
| --- | --- | --- | --- | --- |
| 1 | A qualitative analysis of aspects of treatment that adolescents with anorexia identify as helpful | Zaitsoff, Pullmer, Geller | 2016. | 21 female adolescents interviewed to know what was helpful in their treatment. No mention of gender. |
| 2 | Gender differences found in a qualitative study of a disordered eating prevention program: What do boys have to say? | Gonzalez et al | 2015 | Nothing about role of gender in treatment. |
| 3 | The experience of specialist inpatient treatment for anorexia nervosa. | Smith et al | 2016 | Experience of treatment of women with no consideration to gender issues |
| 4 | Rules of engagement : qualitative experiences | Sly et al | 2014 | Treatment experiences of women with nurses. No mention of gender. |
| 5 | An exploration of the perceptions and experiences of living with chronic anorexia | Fox and Diab, | 2013 | Treatment experiences of 6 women with nothing about role of gender. |
| 6 | Eating disorders patients views on their disorders and on an outpatient service. | Reid et al | 2008.- | 19 women and 1 man interviewed about experiences of treatment. Nothing about gender. |
| 7 | Exploring patients experiences of eating disorder treatment | Sheridan et al | 2016 | Treatment experiences of 14 women, no mention of gender. |
| 8 | Therapeutic engagement: perspective of adolescents | Zaitsoff et al | 2015 | 34 female adolescents views’ on treatment for mental health in general not for eating disorder treatment. |
| 9 | A feminist family therapy research study | Chan | 2006 | Case study of a female about family therapy. |
| 10 | What women in therapy for an ED found helpful? Thesis | Kelley | 2005 | Nothing about gender. |
| 11 | The parent experience of illness treatment and change | Hannon et al | 2017 | Treatment experiences of women in community treatment with no consideration of gender. |
| 12 | Adolescents inpatient treatment for anorexia nervosa | Offord et al | 2006 | Experiences of treatment for AN. |
| 13 | Self admission to inpatient treatment for patients with anorexia | Strand et al | 2017 | Experiences of 15 women and 1 man participating in self-admission program of a specialist clinic. Nothing about gender issues. |
| 14 | You don't have anorexia, you just want to look like a celebrity, | Dimitropoulos et al | 2016 | Perceived stigma about AN, not about gender in treatment. |
| 15 | Adolescents experiences of inpatient treatment for anorexia nervosa | Colton & Pistrang | 2004 | Experiences of an inpatient treatment, not about gender in treatment. |
| 16 | Shame and pride in AN. | Skarderud | 2007 | Role of shame in eating disorders, not about gender in treatment. |
| 17 | Exploring patients experiences of eating disorder treatment | Sheridan et al | 2015 | Treatment experiences of 14 women, no mention of gender. |
| 18 | The individual within a condition | Tierney | 2008 | Treatment experiences of AN, no mention of gender. |
| 19 | Preferred therapist characteristics in treatment of AN | Gullisken | 2012 | Therapist qualities helpful in treatment, no mention of gender. |
| 20 | Treatment of AN in the context of trans sexuality | Ewan et al | 2014 | Case report of a transgender under treatment for AN with no mention of treatment experiences. |
| 21 | ED in a transgendered patient: A case report. | Surgenor & Fear | 1998 | Case report of a transgender under treatment for AN with no mention of treatment experiences. |
| 22 | A life history analysis of a male athlete with an ED. | Papathomas | 2006 | About onset of bulimia not about treatment. |
| 23 | An angel on my shoulder | Wright et al | 2012 | Therapeutic relationship between patient and healthcare professional. |
| 24 | Inside the experience of AN. | Ross & Green | 2011 | Therapeutic experience of 2 women. No mention of gender. |
| 25 | Disciplining the feminine. | Moulding | 2006 | Health care providers not people with a lived experience interview. |
| 26 | Constructing the eating disordered patient. | Malson et al | 2004 | Experience of treatment of 35 women and 1 man. Nothing about gender issues. |
| 27 | Tracing a matrix of gender: an analysis. | Malson et al | 2008 | Nurses account of nursing eating disordered patients. |
| 28 | Shame, pride and ED | Goss | 2009- | Explored shame and shame based responses in ED. Not about gender issues. |
| 29 | Patients evaluation of their former treatment for AN. | Brinch et al | 2009 | Evaluations for treatment. Nothing about gender issues. |
| 30 | Treatment issues and outcome for males with ED | Weltzin | 2012 | Quantitative study |
| 31 | Living with the anorexic voice | Tierney | 2010 | Experience of having AN. Nothing about gender issues. |
| 32 | The quantification of gender. AN and femininity. | Till. | 2011 | Gender identity scales. Not about gender issues in treatment. |
| 33 | Perceptions of thin and other ideals predict treatment outcome. | Thurfjell. | 2006 | Quantitative study to predict treatment outcome. Not about gender issues as experienced in treatment |
| 34 | Being me and being us adolescents. | Lindstedts et al | 2015 | Role of family and therapist in treatment. Nothing about gender issues. |
| 35 | Life as a male anorexic | Drummond | 1999. | Explores lives of male with AN or BN, not about treatment. |
| 36 | Insights into the experiences of treatment | Thapliyal et al | 2017 | Qualitative study but of autobiographies and not of people with clinically diagnosed eating disorders according to diagnostic criteria. |
